# General ward nurses detection and response to clinical deterioration in three hospitals at the Kenyan coast: a convergent parallel mixed methods study

**DOI:** 10.1186/s12912-024-01822-2

**Published:** 2024-03-01

**Authors:** Nickcy Mbuthia, Nancy Kagwanja, Moses Ngari, Mwanamvua Boga

**Affiliations:** 1 Department of Medical Surgical Nursing, School of Health Sciences, Kenyatta University, Nairobi, Kenya; 2 KEMRI Wellcome Trust Research Programme, KEMRI Centre for Geographic Medicine Research Coast, Kilifi, Kenya

**Keywords:** Clinical deterioration, Vital signs, Nurse documentation, Patient safety, Medical surgical nursing, Recognising, Responding, Early Warning Score, Rapid Response System, General ward

## Abstract

**Background:**

In low and middle-income countries like Kenya, critical care facilities are limited, meaning acutely ill patients are managed in the general wards. Nurses in these wards are expected to detect and respond to patient deterioration to prevent cardiac arrest or death. This study examined nurses' vital signs documentation practices during clinical deterioration and explored factors influencing their ability to detect and respond to deterioration.

**Methods:**

This convergent parallel mixed methods study was conducted in the general medical and surgical wards of three hospitals in Kenya's coastal region. Quantitative data on the extent to which the nurses monitored and documented the vital signs 24 h before a cardiac arrest (death) occurred was retrieved from patients' medical records. In-depth, semi-structured interviews were conducted with twenty-four purposefully drawn registered nurses working in the three hospitals' adult medical and surgical wards.

**Results:**

This study reviewed 405 patient records and found most of the documentation of the vital signs was done in the nursing notes and not the vital signs observation chart. During the 24 h prior to death, respiratory rate was documented the least in only 1.2% of the records. Only a very small percentage of patients had any vital event documented for all six-time points, i.e. four hourly. Thematic analysis of the interview data identified five broad themes related to detecting and responding promptly to deterioration. These were insufficient monitoring of vital signs linked to limited availability of equipment and supplies, staffing conditions and workload, lack of training and guidelines, and communication and teamwork constraints among healthcare workers.

**Conclusion:**

The study showed that nurses did not consistently monitor and record vital signs in the general wards. They also worked in suboptimal ward environments that do not support their ability to promptly detect and respond to clinical deterioration. The findings illustrate the importance of implementation of standardised systems for patient assessment and alert mechanisms for deterioration response. Furthermore, creating a supportive work environment is imperative in empowering nurses to identify and respond to patient deterioration. Addressing these issues is not only beneficial for the nurses but, more importantly, for the well-being of the patients they serve.

**Supplementary Information:**

The online version contains supplementary material available at 10.1186/s12912-024-01822-2.

## Background

Failure to rescue is a patient safety and healthcare quality measure, which refers to the delay or failure to detect and respond to clinical deterioration in hospitalised patients, leading to mortality [1, 2]. In-hospital mortality is increasingly being recognised as a measure of service provision quality and a key indicator of patient safety [3, 4]. Although patients inevitably die in the hospital, clinicians should be able to detect the deterioration in the patient early enough and act promptly to prevent death. Early detection and response to the changes in vital signs and physiological parameters in hospitalised patients are essential in reducing the risk of preventable death and preventing unplanned admission into critical care units [5, 6]. Numerous studies have established that there are physiological antecedents before a cardiac arrest occurs in a hospitalised patient, allowing the clinician to identify the deterioration and act promptly [7–10].

Patient monitoring, which involves the assessment of vital signs and physiological changes, allows for early detection of these antecedents of patient deterioration. Subsequently, based on monitoring criteria, the clinician triggers or activates the rapid response team to treat the deteriorating patients before adverse events occur [11]. The most commonly used monitoring and call criteria for rapid response include abnormalities in physiologic measures such as respiratory rate, heart rate, systolic blood pressure, oxygen saturation, acute change in mental status, and the clinician's significant concern about the patient's condition [12]. This can be achieved by either intermittently or continuously monitoring of the vital signs as well as the electrocardiogram (ECG). Regularly monitoring these physiologic measures allows the clinician to identify deterioration and thus act hastily to prevent further deterioration by escalating and communicating the information clearly to the individual or team that shall manage the patient and prevent further deterioration. In addition to monitoring the physiological parameters, it is important to document the findings in a standardised format so as to be able to track the trends in the measures so as to detect the changes in a timely manner. The recommended practice for the documentation of the measure is the use vital signs observation charts that display the information in a way that allows early and speedy identification of deterioration [13].

Escalation of care in most institutions is guided by institutional protocols that clearly outline the actions that the clinician can implement until they are satisfied that the correct response has been achieved. These actions include a change in monitoring frequency, possible modifications of care, a review of the patient by a senior medical officer, seeking assistance from an intensive care specialist, or activation of the Rapid Response System [14]. In addition, a concise, efficient and accurate flow of information about the deteriorating patient is a fundamental component of the escalation of care [14, 15]. The different modes to communicate the deterioration of care include face-to-face communication, overhead announcement within the hospital, mobile and landline telephones, and hospital alarms [16]. Whichever mode is used to communicate deterioration, it is critical to use a common language using a structured communication tool to ensure a timely response.

In the critical care units (CCUs), patient's vital signs are continuously and closely monitored with monitoring equipment and technologies, and therefore detection of deterioration and response is timely. Additionally, the CCUs are well equipped with resuscitation equipment and are adequately staffed with staff trained in responding to changes in patient deterioration. Nevertheless, in the general wards where a majority of instances of clinical deterioration take place, patient assessment involving the measurement of vital signs is conducted at irregular intervals. Research indicates that in these general wards, nurses might monitor vital signs anywhere from every four hours to as infrequently as every 12 h [17, 18]. The frequency of monitoring depends on factors such as nursing workload, the type of patients being treated, and the availability of resources[19, 20]. Therefore, it depends on the clinicians' ability to recognise and respond to the deterioration.

In Kenya, like most low and middle-income countries, patient complexity is increasing because of higher morbidity due to non-communicable diseases, road traffic accidents, and a longer life expectancy. This has led to a higher demand for critical care services as compared to high income countries, yet the availability of these services is limited [21–24]. This has therefore led to acutely ill patients being managed in the general medical and surgical wards located in the secondary referral facilities. The Kenyan public health system is organised in four tiers with six levels namely, the community level (level one); primary healthcare (dispensaries and health centres-level two and three respectively); secondary referral facilities (levels four and five) and tertiary referral health services (level six) [25]. In-patient services which include the general and specialised wards are offered in levels four to six. Based on policy guidelines these hospitals should have a range of health providers including medical specialists, nurses, clinical officers, radiographers and lab personnel and equipment that can support prompt identification of clinical deterioration [26, 27]. However, most of the existing facilities (83%) do not meet the health infrastructure norms and standards requirements for their level. Further studies have shown that there are significant staffing gaps with insufficient numbers of specialists across many level four to six hospitals [28, 29].

Due to the critical role that nurses play in early deterioration identification, failure to rescue may be very sensitive to nursing care. According to Mushta et al. (2018) [30], failure to rescue as a nurse-sensitive indicator includes failure to recognise changes in patient condition, failure to escalate the changes and inadequate decision-making. Patient assessment and monitoring is a fundamental nursing competence; therefore, nurses carry the highest responsibility in detecting and responding to patient deterioration. However, research shows that nurses fail to recognise and respond timely to patients' deterioration [31–33].

Vital signs monitoring is fundamental to nursing, whereby the nurse must assess, document, and interpret vital signs promptly and escalate any abnormal values. However, research shows that monitoring, documentation, and reporting of vital signs are often irregular, incomplete, and not used to make clinical decisions [20, 34, 35]. Documented factors that contribute to the failure of a nurse to recognise and respond include the lack of knowledge and skills in the detection of warning signs, poor or absent monitoring of the patient's vital signs, difficulty in prioritising competing demands with increasing workload and fear of reporting the deterioration [36–40]. Notably, most of the literature on the general ward nurses' issues and challenges in detecting and escalating patient deterioration has been generated in high-income country settings, with little literature from low and middle income countries. We did not identify any studies reporting on the Kenyan context. Therefore, this study aimed to examine the general ward nurses' vital signs documentation practices in the deteriorating patient and explore the factors influencing their ability to detect and respond to clinical deterioration in three Kenyan hospitals.

## Methods

### Design

A convergent parallel mixed methods design was utilised to achieve the objectives. We used this design because it allowed the collection and analysis of both quantitative and qualitative data with the aim of achieving triangulation whereby the qualitative results corroborated the quantitative results thus providing us with a more comprehensive understanding of our research problem [41]. A retrospective descriptive design was used to retrieve quantitative data from medical records of patients who had suffered a cardiac arrest and died in the previous year in the study hospitals' general medical and surgical wards. Specifically, the study sought to determine the extent to which the nurses monitored and documented the patients' vital signs 24 h before a cardiac arrest (death) occurred. Additionally, qualitative data was collected by conducting in-depth interviews with nursing staff, including ward managers, to understand better the factors that influence the ability to detect and respond to clinical deterioration. Data from the two methods were analysed separately, and the results were compared and related. Each type of data was used to validate the other during the interpretation, creating solid foundations through which we could better achieve the study objectives [42].

### Setting

The study was undertaken in the adult medical and surgical wards of three purposefully selected hospitals in Kenya's coastal region. We identified hospitals with prior contact or linkage with the research team to minimise trust concerns related to our data collection given the sensitive nature of our research topic. These linkages were based on teaching and research relationships and were useful to gain access to study participants. To reduce bias, we adopted method triangulation where we adopted document review and in-depth interviews and data source triangulation by conducting interviews with respondents across three hospitals. One hospital in Mombasa County was a Level Five hospital (Hospital A), while the other two were Level Four hospitals (Hospital B and C) in Kilifi County. The three hospitals are relatively homogeneous in their organisational structure, resource availability, and the populations they serve however, Level Five hospitals offer a more comprehensive array of specialised curative services than level 4 hospitals [43].

### Sample and Sampling technique

#### Nurses' documentation review

The sample comprised the records of patients who suffered a cardiac arrest and died in the adult medical and surgical wards in 2019. We decided to review the records of these patients because it is well documented that patients frequently display physiological changes as much as 24 h before a cardiac arrest and death occur [7, 9]. Therefore, we included all records from male and female patients 18 years and older and those admitted for more than 24 h between January and December 2019 from which we screened 405 records from the three hospitals.

#### Key informant interviews

We used purposive sampling technique to get a sample of registered nurses working in the adult medical wards and the surgical wards in the three hospitals. We included in the interviews nurses who had worked in the adult medical and surgical wards in the study hospitals for at least six months, carried out clinical duties, and were willing to be interviewed. We therefore carried out interviews on twenty-four nurses which included bedside nurses, and ward managers. Ward managers were included because in the three hospitals, these nurses also provided bedside nursing care as well as ward administration responsibilities. In the administrative role, the ward managers were usually able to identify nurses’ needs and possible areas for support and resources required to facilitate prompt identification and response. We also interviewed two hospital management nurses so as to get a deeper understanding of the organisational factors influencing the recognition and response to clinical deterioration by the nurses.

### Data collection procedures

#### Documentation review

Data collection took place between August 2020 and August 2021. In hospital B, a list of all the deaths that occurred in the adult medical and surgical wards in 2019 was obtained from an electronic database. A systematic sampling technique was used to obtain the sample. It was possible to obtain the patient's admission number from the database, which was then used to retrieve the patient's file in the medical records department. Hospitals A and C had no databases of the deaths available. Therefore, with the assistance of the medical records personnel, the data collectors retrieved all the files of the patients who had died in the respective hospitals in 2019. Subsequently, they selected the files from the adult medical and surgical wards from which they randomly selected and analysed files for each month in 2019.

To examine if, which, where, and how physiological parameters of the patients were documented before patient deterioration, the sampled records were reviewed for the nursing documentation of six vital signs 24 h before death. The vital signs were respiratory rate in breaths per minute, systolic blood pressure in millimetres of mercury (mmHg), oxygen saturation as a percentage, heart rate in beats per minute, temperature in degrees centigrade, and consciousness level. According to the literature, to detect patient deterioration, these are the minimum physiological parameters that trigger the nurse to call for help, and they should be monitored at least every 4 h [12, 44].

Data were collected using a predesigned tool developed by the researchers based on recommendations from literature and guidelines on documentation of patients' physiological observations [45]. In the data collection tool, the research assistants first identified which document the vital signs were retrieved from, either the vital signs observation chart or the nursing notes (Kardex). According to the literature, acute patients' vital signs should be monitored every four hours unless the patient's condition or specific treatment requires more frequent measurements [46]. Therefore, the data collection tool also captured the last six vital signs documented by the nurse in the previous 24 h before death occurred. If none of the vital signs had been recorded in the 24 h preceding the cardiac arrest, the last recorded vital sign(s) was captured, including the time it was recorded.

#### Key informant interviews

We conducted in-depth in-person interviews in each hospital to better understand the factors that influence the ability to detect and escalate clinical deterioration. We developed two separate interview guides for the general ward nurse and for the ward and hospital managers. We used the guides to explore the ward nurses' experiences in caring for the deteriorating patient, the challenges in detecting deterioration, their actions when a patient deteriorates, and the communication practices for escalating the deterioration. To learn about factors influencing ability to detect and respond to clinical deterioration we asked questions about the nurses’ views on the support and resources available to them. These questions including the probes for them are included in the interview guides in Supplementary File 1. In addition, examples of clinical deterioration were used to prompt the recall of similar situations experienced by participants. The scenarios aimed to trigger reflection on personal experiences.

We sought informed consent before conducting interviews. The interviews were conducted in offices within the nurses' respective wards, where we anticipated few interruptions. However, because the interviews were conducted during regular shift hours, the nurses' colleagues were informed of the locations in case of any urgent situations. All interview sessions lasted approximately 20–30 min and were recorded using an audio recorder. The interviews took place in an office setting that guaranteed privacy and an acoustically conducive environment. The interviews were continued in each hospital until data saturation was achieved. Since data collection occurred during the COVID-19 pandemic, we adopted prevention measures such as wearing masks and social distancing during the interviews.

### Data analysis

#### Analysis of the documentation

All quantitative data were extracted from patient records using a standard tool and entered into the Epidata database. All categorical patient characteristics were reported as frequencies and their respective percentage. Age in years was reported as median and interquartile range (IQR) because it was skewed. Documentation for each vital event was reported as the number of patients with a vital event documented at each time point. Statistical analyses were conducted using Stata (Version 17.0, College Station, Texas, USA).

#### Analysis of the interviews

For the qualitative data, all audio recordings and field notes were maintained in a secure location. A professional transcriber transcribed the audio recordings into an MS WORD document. The researchers listened to the recordings and compared them with the transcription to ensure accuracy and enhance familiarisation with the data. We adopted a thematic analysis approach, as described by Braun & Clarke, 2006 [47], where we sought to identify patterns in the reviewed transcripts. Our analysis was deductive, driven by the research objectives and our focus on nurses' experiences of patient clinical deterioration. The analysis process involved familiarisation with the data, generating initial codes, searching for themes, reviewing themes, defining themes and writing up the report [47, 48]. The process, though described step-wise, was iterative.

### Credibility and Trustworthiness

A wide range of respondents was identified for this study to provide as much depth and understanding as possible on the practices and experiences of identifying and responding to clinically deteriorating patients. These included speaking with nurses working across different in-patient wards within the study hospitals, and including nurse managers, majority of whom also have had clinical service delivery roles. Further we utilised multiple data collection methods to support triangulation of our findings and enhance exploration of our research question. The first and second authors led the qualitative analysis with input from the rest of the study team. After the first round of coding, emerging categories were shared with the study team for discussion. This provided input that shaped and refined the thematic framework, providing a form of peer debriefing [49]. To achieve dependability (showing that the findings are consistent and could be repeated), we endeavoured to maintain a clear audit trail of the research process by adopting a systematic research approach using data collection and study methods that meet widely accepted standards for qualitative research. We have also provided the rationale for methodological choices including our choice of study respondents, data collection and analysis approaches.

### Ethical considerations

Ethical review and approval were obtained from the Pwani University Ethics Review Committee (ERC/PU-STAFF/001/2020). A research permit to conduct the study was obtained from the National Commission for Science, Technology & Innovation (NACOSTI/P/20/4890). Upon ethical approval, permission to collect data was granted by the respective hospital boards after assurances to adhere to the COVID-19 preventive measures to protect the study participants and the data collectors. Interview participation was completely voluntary. The participating nurses were provided with written information about the research and their rights to the study. The participants were requested to sign and date the informed consent form upon agreement.

## Results

### Quantitative analysis results

#### Patient characteristics

A total of 405 records were reviewed, 186 (46%), 135 (33%), and 84 (21%) in Hospital A, Hospital B, and Hospital C, respectively. Their median [IQR] age was 54 [36 to 68] years. Two hundred and forty-two (60%) were male. The most frequent diagnoses identified from the reviewed patient files were cancer 41 (10%), Cerebrovascular accident 30 (7.4%), Heart failure 27 (6.7%), Tuberculosis 25 (6.2%) and Meningitis 24 (5.9%) shown in Table 1.

**Table 1:** Patient Characteristics

	**A (*N* = 186)**	**B (*N* = 135)**	**C (*N* = 84)**	**Overall (*N* = 405)**
Sex				
Male	120 (65)	78 (58)	44 (52)	242 (60)
Female	66 (35)	57 (42)	40 (48)	163 (40)
Age in years				
Median [IQR]	48 [35-65]	60 [37-70]	55.5 [42-70]	54 [36-68]
Leading primary diagnosis				
Cancer	22 (12)	14 (10)	5 (5.9)	41 (10)
Cerebrovascular accident	10 (5.4)	13 (9.6)	7 (8.3)	30 (7.4)
Heart failure	10 (5.4)	10 (7.4)	7 (8.3)	27 (6.7)
Tuberculosis	14 (7.5)	9 (6.7)	2 (2.4)	25 (6.2)
Meningitis	8 (4.3)	13 (9.6)	3 (3.6)	24 (5.9)
Chronic Kidney Disease	13 (7.0)	5 (3.7)	6 (7.1)	24 (5.9)
Pneumonia	9 (4.8)	9 (6.7)	4 (4.8)	22 (5.4)
Sepsis	14 (7.5)	3 (2,2)	4 (4.8)	21 (5.2)
Anaemia	6 (3.2)	9 (6.7)	5 (6.0)	20 (4.9)
Gastroenteritis	9 (4.8)	3 (2.2)	8 (9.5)	20 (4.9)
Head Injury	15 (8.1)	3 (2.2)	1 (1.2)	19 (4.7)
Diabetes Mellitus	7 (3.8)	3 (2.2)	7 (8.3)	17 (4.2)
Liver disease	5 (2.7)	6 (4.4)	2 (2.4)	13 (3.2)
Hypertension	6 (3.2)	4 (2.9)	3 (3.6)	13 (3.2)
Acute Kidney Injury	3 (1.6)	7 (5.2)	2 (2.4)	12 (3.0)
Others	35 (19)	24 (18)	18 (21)	77 (19)

Results are *N* (%) unless specified, *IQR* Interquartile range.

#### Patient deterioration nursing documentation

**Fig. 1:**
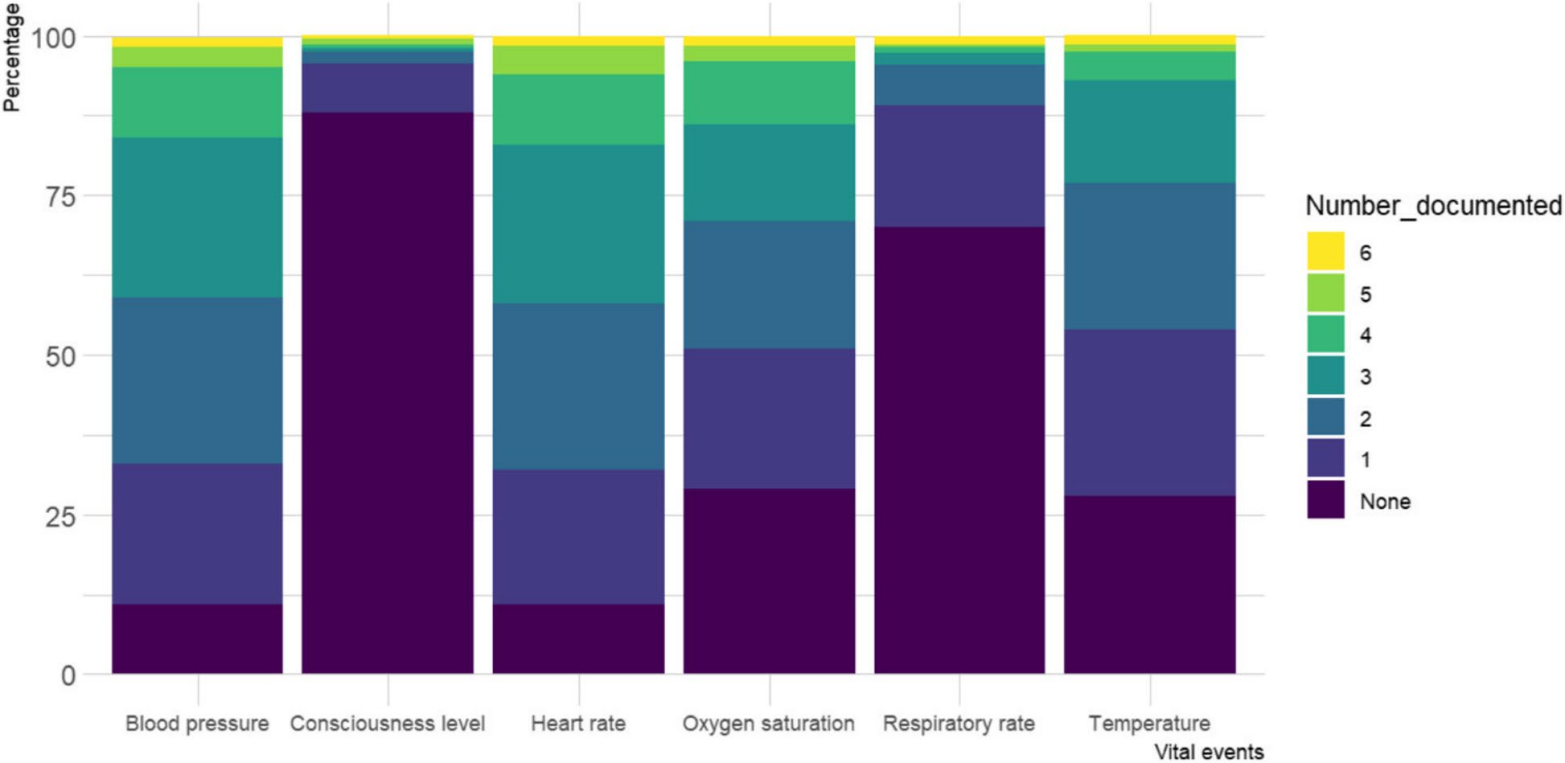
A stack bar chart showing proportions of vital events documented at the six-time points

Of the 405 patient records reviewed, the vital signs were documented in the nursing notes (Kardex) and vital signs observation chart among 391 (97%) and 14 (3.5%), respectively. During the 24 h prior to patient deterioration/death reviewed, 283 (70%) patients had no respiratory rate documented, 77 (19%) had only one documentation of respiratory rate, while only 5/405 (1.2%) had documentation for all the six-time points. There was no heart rate documentation among 45 (11%) patients, while 87 (21%), 107 (26%) and 101 (25%) had one, two and three documentations, respectively. The temperature was not documented among 115 (28%) patients, while it was documented once, twice and thrice among 104 (26%), 92 (23%) and 65 (16%) patient files, respectively. There were 43 (11%) patient files with no blood pressure documentation, and it was documented among 91 (22%), 107 (26%) and 102 (25%) patients once, twice and thrice, respectively. Oxygen saturation was not documented among 116 (29%) patients but was documented once, twice and thrice among 91 (22%), 80 (20%) and 62 (15%) patients, respectively. Almost nine in every ten patients (*N* = 356, 88%) had no documentation for the level of consciousness but was documented once, twice and thrice among 31 (7.7%), 7 (1.7%) and 3 (0.7%) patients, respectively, as shown in Table 2. Less than 2% of patients had any vital signs documented for all the six-time points; respiratory rate (*N* = 5, 1.2%), heart rate (*N* = 6, 1.5%), temperature (*N* = 6, 1.5%), blood pressure (*N* = 6, 1.5%), oxygen saturation (*N* = 5, 1.2%) and level of consciousness (*N* = 2, 0.5%) as illustrated in Fig. 1.

**Table 2:** Patient deterioration nursing documentation review

Vital events	Number of final six vital signs documented						
	None	1	2	3	4	5	6
Respiratory rate	283 (70)	77 (19)	26 (6.4)	8 (1.9)	4 (0.9)	2 (0.5)	5 (1.2)
Heart rate	45 (11)	87 (21)	107 (26)	101 (25)	45 (11)	14 (3.5)	6 (1.5)
Temperature	115 (28)	104 (26)	92 (23)	65 (16)	18 (4.4)	5 (1.2)	6 (1.5)
Blood Pressure	43 (11)	91 (22)	107 (26)	102 (25)	43 (11)	13 (3.2)	6 (1.5)
Oxygen Saturation	116 (29)	91 (22)	80 (20)	62 (15)	42 (10)	9 (2.2)	5 (1.2)
Level of consciousness	356 (88)	31 (7.7)	7 (1.7)	3 (0.7)	2 (0.5)	4 (1.0)	2 (0.5)

Results are *N* (%).

### Qualitative analysis results

#### Participants characteristics

We conducted 24 interviews with respondents across the three study hospitals. Of these 24, there were two senior hospital management nurses and six ward managers. All the nurse ward managers reported carrying out clinical nursing duties and ward administration responsibilities. Table 3 below summarises the participants' characteristics related to their gender and how long they had provided nursing services.

**Table 3:** Interview respondent characteristics

Characteristic	Hospital A (*N* = 10)	Hospital B (*N* = 8)	Hospital C (*N* = 6)
**Overall duration as a nurse (years)**			
0–5	1	4	2
6–10	2	0	1
11–20	4	3	0
21–30	3	1	1
> 30	0	0	2
No. of years working in current ward*			
6 months-1 year	5	1	1
2-3 years	1	4	3
4-5 years	2	2	0
> 5 years	2	0	1
**Gender**			
Male	1	3	2
Female	9	5	4
Nurse roles			
Ward manager	3	2	2
Ward nurses	7	5	4
Nurses in senior management		1	1

*We were not able to access a nurse in senior management from Hospital A

#### Ward characteristics

Table 4 summarises some hospital and ward characteristics that appeared to influence the extent to which nurses could monitor the patients and document their findings. In hospitals B and C, the medical and surgical patients were nursed in the same ward. The wards were distinguished as male and female, with the female wards comprising patients with medical, surgical (general surgical and orthopaedic) and gynaecological conditions. The male wards (hospitals B and C) also admitted medical and surgical patients. Further, some admitted patients had psychiatric conditions in hospital C. Hospital A had separate male and female medical and surgical wards. As a result, hospital A respondents were more in number (Table 3) compared to Hospital B and C. Hospital A had an Intensive Care Unit (ICU) with a capacity of 15 beds (Table 4), where patients needing critical care from Hospitals B and C were also referred. The unavailability of ICUs in Hospital B and C required that patients in need of critical care were referred to Hospital A. This posed as a challenge because the critically ill patient continued being managed in the general wards if the ICU in Hospital A was full, as quoted by one of the participants:

*The person who is receiving the call will tell you my ICU is full. What will you do after you hear that? How will you verify if ICU is full? You have very critical patient who really, really needs ICU, needs ventilation…The present condition is going down you end up risking with fluids, with medication, you can put oxygen and that's all. As we wait like 3,4 hours you call again you ask. Patients end up staying for like for 3, 4 days even without going for an ICU. So, we lose patients just like that. (B006)*

**Table 4:** Ward characteristics in study hospitals

Ward Characteristics	Hospital A	Hospital B	Hospital C
Type of patients admitted into wards	Separate medical and surgical wards for female and male patients	Mixed female ward (admitting, medical, surgical and gynaecological patients)	Mixed female ward (admitting, medical, surgical and gynaecological patients)
ICU or HDU in a hospital	ICU present	No ICU	HDU present
No. of nurses working in the study wards	Male surgical: 22	Male ward: 10	Male ward: 10 (but two away on study leave)
	Female surgical: 11		
	Male medical: 21	Female ward: 11	Female ward: 8 nurses (2 away on leave)
	Female medical: 20		
The bed capacity of the study ward	Male surgical: 70	Male ward: 34 patients	Male ward: can hold up to 60 patients
	Female surgical: 40		
	Male medical: 72	Female ward: 32 patients	Female ward: 48 patients
	Female medical: 80		

*Abbreviations*: *ICU* Intensive Care Unit, *HDU* High Dependency Unit

### Factors influencing nurses' ability to detect and respond to clinical deterioration

Interview respondents acknowledged that they faced challenges in detecting and responding to clinically deteriorating patients in the ward. Five broad, interrelated themes related to detecting and responding promptly to patient deterioration emerged from the analysis of qualitative interviews. These were *insufficient monitoring of vital signs and inadequate response to clinical deterioration linked to limited availability of equipment and supplies*, *staffing conditions and workload*, *lack of training and guidelines*, *communication and teamwork among healthcare workers*. These are discussed in turn below.

#### Insufficient monitoring of vital signs and inadequate response to clinical deterioration linked to limited availability of equipment and supplies

The qualitative results were consistent with the findings of the document review related to gaps in vital signs documentation. Across all three study hospitals, interview respondents reported inconsistently taking vital signs for patients admitted to the wards. These inconsistencies, linked to poorly functioning and limited availability of equipment for monitoring patients, also manifested in poor documentation of patients' vital signs, as illustrated in the quote below:

*And the biggest challenge is that we don't have the apparatus at hand. For example, if we are depending on one monitor to observe all the patients and then monitor breaks down. Like for now, you go borrowing from another ward, you find that it is being used. This really disturbs the documentation especially when it comes to vitals. So, you can find a patient may be observed maybe twice, thrice maybe a patient who has stayed for like 3 or 4 days which is a quite an ugly picture (A003)*

Instances of poorly functioning equipment included the failure of the digital blood pressure machines and thermometers. Respondents reported that this equipment functioned well if their use was limited to a few patients. However, as highlighted in Table 4 above, the wards where the respondents worked frequently had high patient numbers, for whom the nurses only had one digital thermometer and blood pressure machine for patient monitoring. It was also common for other monitoring equipment, such as blood sugar machines, to sometimes lack batteries. There were also instances where monitors had been present in the wards but were not in use because they had not been repaired or replaced. Another type of equipment which was reportedly absent in one of the study hospitals was a pulse oximeter. In this hospital, the respondents reported relying only on observations of respiratory distress to administer oxygen and held the view that they perhaps were giving oxygen too late because they were unaware of the patient's oxygen saturation. For example, one respondent stated that,

*In case a patient starts gasping, that's when we put them on oxygen because we don't have things like oximeter that we can be able to monitor oxygen saturation. I would say it's mostly when the patient is going down, they are gasping or difficulties in breathing then we start them on oxygen. (B003)*

Despite these challenges, there were efforts to closely monitor patients judged to be severely ill. Most of these patients were placed in the acute rooms of the different wards to enable the nurses to quickly detect changes in the patient's conditions. In addition, when there were students in the ward, they were utilised to closely monitor the patients as they learnt. However, because students were still in training, they were not always able to quickly detect a deteriorating patient and communicate this to the nurse in good time.

Regarding the low availability of equipment and supplies, many respondents highlighted that oxygen, commonly used to respond to deteriorating patients, was often not readily available. In Hospitals B and C, some of the beds in the acute bay had oxygen ports for piped oxygen. However, if more than four patients, for example, in the case of Hospital C, needed oxygen, then an oxygen concentrator or oxygen cylinders would have to be used. However, the oxygen concentrator shared between the male and female wards in Hospital C had been broken down for over six months without repair. They, therefore, had to borrow from either the paediatric ward or High Dependency Unit (HDU). In Hospital A, they mainly used oxygen cylinders but reportedly experienced delays in connecting the oxygen as they tried to move the heavy oxygen cylinders:

*We don't have concentrators, we don't have oxygen, and there is a patient who needs oxygen, and it's not there. We don't even have a concentrator (C003)*

*There is also a challenge there in terms of because these cylinders sometimes we run out of oxygen and you find we go to another ward cylinders are in use, cylinders are heavy these big cylinders. So you find a nurse is not able to move that cylinder (A001)*

Other equipment-related factors that hindered prompt response to deteriorating patients included the incomplete nature of the emergency trays in some wards. One respondent reported:

*Yes, we had a crash cart but we have never been able to manage the crash cart. Because you see, one, people still have that habit of picking drugs. So today you can refill the crash cart then you find within a minute you are not seeing those drugs maybe by the end of the day no single drugs because people pick, pick and they don't replace, there is a lack of discipline in a way someone can just come dashing because maybe a patient has changed condition in another ward maybe a medical ward looking for a certain drug, picks and go (A003)*

Respondents also highlighted that having one bag valve mask (BVM) per ward meant that there was a delay when a patient required resuscitation, especially if the BVM was not functioning well. In such cases, nurses had to borrow from another ward. Another inadequate supply that hindered prompt responses was Personal Protective Equipment (PPE). This was particularly relevant for nurses who were managing COVID-19 patients.

#### Staffing conditions and workload

Across all the study hospitals, most respondents felt that the adult medical and surgical wards were grossly understaffed, making monitoring and detecting deteriorating patients difficult. As noted above, an acute bay was supposed to help nurses quickly detect when patients' conditions changed. However, this quick identification was limited in most cases because the nursing shifts were often covered by few nurses, making it challenging to do timely observations of vital signs. For example, one respondent from hospital B observed:

*Even though the male and female wards have acute areas. The only benefit of the acute areas is that the nurse is near the patients. However, it is difficult to do close monitoring when the nurse is working alone and the patients are many... because I have said sometimes we have a capacity of 45. So it won't be easy for you to be checking vitals maybe every 2 hours. (B005)*

In the afternoon and night shifts, nurses commonly worked alone, with the support of students. The morning shifts were reportedly better staffed, as about two to three nurses reported to work. Most respondents felt that it was easier to detect deteriorating patients during the morning shift. During the shifts where a nurse was working alone, it was reportedly not uncommon for nurses to find out from relatives that patients were deteriorating as they did other procedures in the hospital wards. In fact, nurses across all three hospitals considered relatives a resource who could inform them when patients deteriorated.

*And sometimes because of the shortage, we are not able to identify as quick as we can. You can imagine when you are alone in the ward, you are giving treatment on the other side. So in the most cases it's the relatives who calls you. They call you, this one has deteriorated and then we notice. (C003)*

*So you are only getting something you are told by the caretaker this patient has deteriorated. And by the time you are being told that, you realised that it has gone to the extreme end. You don't get to intervene early enough. When you go and see this patient the general condition has already deteriorated to a further extent that the measures you take maybe you are trying to resuscitate this patient anaenda tu hivo [dies]. Nevertheless, you try. (B006)*

*When the nurse is alone and you are on the other side and you left patient A, you have maybe 30 patients, like now we have 27 and the night nurse is alone, so she left patient A and now she is doing patient 25, and this A changes condition, unless the relatives call you, you will not know. By the time you go there, maybe it will be too late. So I think the nurse/patient ratio plays a big role. (A006)*

The challenges with staffing also hindered effective response to a deteriorating patient, particularly one who required resuscitation, as described below by one of the respondents:

*You are alone, and the patient has changed; the one you have is a student. Some of the students they are very junior, they don't know anything, tell me, whom will you call? And here you know resuscitation is teamwork. Teamwork of around 3, 4 people together working. When you are alone it becomes very difficult to do all those procedures alone. You need someone to record [vital signs], the other one do cardiac massage...If there is no that teamwork, the patient becomes zero because when you are alone, you can't do. (A005)*

When responding to a deteriorating patient, staffing was not only a nursing issue; it also seemed to cut across to other healthcare professionals who worked in the ward. Doctors especially were commonly few and busy, and the respondents reported delays when they called them. At night, across the three study hospitals, the doctors were not available in the wards and were commonly in casualty admitting patients. In Hospitals B and C, one Medical Officer (MO) intern circulated across the casualty department, theatre, and wards.

#### Lack of training and guidelines

Across all the study hospitals, most of the nurses had not been trained in Cardiopulmonary Resuscitation (CPR), Basic Life Support (BLS) or Advanced Cardiac Life Support (ACLS) despite caring for the acutely ill patients in the wards. Instead, most nurses relied on their pre-service training to detect and respond to deteriorating patients.

*Very few people have gone through BLS, ACLS. Personally, I haven't done BLS, ACLS because it's time and money… I don't know but 90% of the people who are there have not done the BLS and ACLS. (B006)*

*I have not done any [BLS or ACLS training]. I did it in college, and that's what I am surviving with…(C004)*

In addition, there were no existing policies and guidelines that were observed or reported on in relation to processes for detecting and responding to deteriorating patients. The exception was in Hospital A, where there were posters with some guidance on the ward notice boards, but the nurses in these wards reported that no training had preceded the introduction of these posters.

#### Communication and Teamwork among the healthcare workers

Experiences of communication varied across the three hospitals. For example, in Hospital A, an alert system that utilised colour codes (e.g. code blue, code red) had been introduced. There were posters at the ward level describing the response process when a patient was noted to be deteriorating. This process required calling through the operator, who would then call the doctors. Unfortunately, few study respondents from Hospital A understood this alert system, and many reported that they had not been trained in its use. As a result, it was hardly utilised. In hospital B, the ward nurse was expected to call the Medical officer (MO) intern to notify them and request their support if there was a deteriorating patient. The intern would then escalate the issue depending on his ability to address the patient's deterioration. In hospital C, there was a long chain of communication in which the ward nurse was required to call the nurse covering the hospital, who would then call the MO intern or the MO to support a response to a deteriorating client. This long chain of communication inevitably slowed down the response to the deteriorating client, as illustrated in the quote below:

*Communication is another issue. I have to call the nurse who is covering the one who has the phone, to call the MO. You see that. It's an emergency, I call somebody to call for me the MO. You can imagine the one I am calling she is in the office, maybe for example at night or in the afternoon...I call her, I have a patient who is gasping, let me call the MO intern, MO intern comes, she feels I can't handle this alone, let me call the MO. You see the chain. Is that an emergency? (C003)*

Notably, the challenges described above were experienced mainly during the night and afternoon shifts and the weekend when there were few staff in the hospital. The respondents noted that during the morning shift, doctors and clinical officers were more available in the ward, and sometimes it was not necessary to make a phone call to reach them to communicate that a patient was deteriorating. Regarding what was communicated when a call was made, while the respondents acknowledged that there were no written-down guidelines that described what should be said, they reported describing the patient's details, the diagnosis, and the most recent vital signs of the patient, including what actions had been taken by the nurse who was calling.

Because of the reported staffing challenges, teamwork appeared to be lacking in ensuring timely responses to deteriorating patients. This was illustrated by reports of certain doctors being too focused on their patients that they did not participate when there was a call for resuscitating a patient that they did not admit:

*Patient is deteriorating from theatre, now I have called the MO. The MO is referring me to the surgeon. Then the surgeon is not picking the phone and I have called over several times, I called the nurse covering. So went back to the MO, so he insisted call the surgeon, but we insisted, please for the sake of the patient, please come and assist us in resuscitating the patient. So we had to apply our own… so we had to resuscitate the patient on our own. (B002)*

## Discussion

This study sought to understand an under-investigated issue in the Kenyan setting. Nurses' identification and response to a deteriorating patient is a critical role that needs to be further illuminated and improved upon to reduce unplanned admission to the few available critical care units as well as to reduce morbidity and mortality. Therefore, this study sought to find out how nurses documented the patients' vital signs 24 h before a cardiac arrest (death) and to understand better the factors that influenced the ability to detect and respond to clinical deterioration. As a result, we identified five central influencing factors that hinder the nurses' ability to detect and respond to patient deterioration. These were the insufficient monitoring of vital signs, availability of equipment and supplies, staffing conditions and workload, lack of training and guidelines, and communication and teamwork amongst healthcare workers.

This study found that vital signs monitoring and documentation by the nurses in the general wards was suboptimal and below the recommended standards of vital signs observations for an acute patient. This finding was consistent both in the document review and from the reports by the interviewees. From the document review, we found that less than 2% of the reviewed patient's files had complete documentation of the six vital signs in the 24 h prior to death. In addition, 70% of the patients did not have any recording of the respiratory rate 24 h prior to death despite it being the more accurate predictor of clinical deterioration [50, 51]. Furthermore, the documentation of the vital signs was primarily done in the nursing notes and not in the vital signs observations chart, therefore, limiting who could access the information. It is important to note that this study only considered the vital signs documented in the nursing notes and observation charts. Therefore, the vital signs may have been taken but not documented and thus could explain the findings in this study. However, these findings are consistent with reports from studies that show that vital signs monitoring and documentation are often incomplete, with the respiratory rate being the lowest monitored vital sign [19, 52, 53]. Identifying signs of deterioration necessitates consistent and precise monitoring of vital signs, along with thorough documentation and presentation of measurements in a manner that enables the detection of any deviations from the expected norms. The absence of these practices may result in delays or an inability to promptly recognise and address clinical deterioration among hospitalised patients, thereby compromising their safety. Conditions that could have been effectively managed with swift intervention might escalate into emergencies, jeopardizing the safety of the patient. Furthermore, regular monitoring and documentation of vital signs play a pivotal role in detecting patterns over time. Failing to spot trends, whether indicative of gradual deterioration or improvement, can impact decisions regarding treatment and patient outcomes, as well as lead to extended hospital stays.

A cross-cutting challenge that the study participants reported was the unavailability of equipment and supplies to enable them to detect and respond to clinical deterioration. This included the lack of monitoring devices, which were not functioning correctly where available. Additionally, they indicated a lack of resuscitation equipment and supplies and a lack of PPEs in the face of COVID-19. However, the most glaring issue was the lack of oxygen supply in the general wards reported across all categories of nurses in all three hospitals. Where available, it was available in oxygen cylinders that required the nurse to move the heavy cylinder from one location to another hence delaying the response to deterioration. The unavailability of oxygen in the healthcare facilities in Kenya became a significant focus during the COVID-19 pandemic, with reports of many hospitals lacking reliable oxygen access [21, 54]. A dependable source of oxygen is necessary to administer oxygen therapy, which can be obtained through various means. They include oxygen tanks that are filled at a facility, oxygen concentrators that extract oxygen from the surrounding air, oxygen plants that distribute oxygen via pipes or tanks, and liquid oxygen provided by a specialised gas plant and stored at high pressure on the premises [55]. However, studies show that ensuring a steady and appropriate oxygen supply in the lower and middle countries remains challenging [56–59]. This is not only hampered by factors such as the use of low-quality equipment that is not well-maintained but also by broader systemic issues, such as an unsteady power supply, limited healthcare workforce, and insufficient funding for healthcare [60, 61].

Studies show that vital signs monitoring and documentation takes a considerable amount of nurses' time, which tends to increase depending on whether the observations are being done 6-hourly, 4-hourly or more [62–64]. The documentation burden is further exacerbated by overwhelming nursing workload and poor staffing as we found in this study. For example, Table 1 highlighted patients with a wide range of diagnoses in the study wards, while Table 4 highlighted significantly low numbers of nurses against a high patient census. Poor staffing affected nurses and doctors in all three hospitals and was a common factor influencing the detection and response to patient deterioration reported by ward nurses who experienced it first hand and confirmed by the nurse managers. These findings are consistent with those from other studies in Singapore and the United Kingdom, where poor staffing led to increased workloads and therefore was a barrier to early detection and response to the clinical deterioration [20, 65]. The World Health Organization acknowledges the shortage of healthcare workers (HCWs) globally but reports that nurses are the most affected cadre of HCWs. The African region is among the most affected regions, with an estimated shortage of 4.2 million HCWs as of 2013 [66]. In Kenya, several studies have highlighted understaffing problems [67–69]. Our study adds to this literature by illustrating how sub-optimal staffing at the ward level influences the capacity of nurses to identify deteriorating patients and respond timely. The challenges of staffing coupled with overwhelming workloads suggest that support for nurses is critical to achieving prompt identification of and response to deteriorating patients. Recent literature proposes that such support could be provided by engagement of nursing assistants to whom nurses can delegate certain roles Fitzgerald et al. [70]. This could potentially free up nurses to identify and respond to deteriorating patients more promptly. However, these authors also note that formal introduction of such a role in the Kenyan health system would require a greater evidence base than is currently available to inform acceptability and the right skill mix of nurses and nursing assistants that would be required to ensure patient safety and quality of care [70]. The Singapore study by Chu et al. found that even with nursing assistants available in their study setting, there were incidents of missed care linked to delegation challenges between nurses and nursing assistants [65]. This illustrating that additional support for nurses would require a multi-faceted approach that goes beyond basic life support training to include appropriate delegation.

Effective communication is crucial for quickly identifying, escalating, and responding to clinical deterioration. Additionally, it is essential that there are laid down processes to enable the clinician to communicate the information clearly, logically and precisely [71, 72]. Furthermore, to guarantee safe and reliable care of the deteriorating patient, effective communication requires clinicians' teamwork based on mutual respect, problem-solving and sharing of ideas [73]. But as reported by the nurses from all three hospitals, they perceived that there was inadequate communication between clinicians, a lack of a process for ensuring timely management when patients deteriorate, and a low level of teamwork and collaboration amongst the different cadres of healthcare workers in agreement with findings from other studies [38, 74–76]. The importance of effective communication and teamwork cannot be understated in a healthcare system that is becoming increasingly complex, fragmented and with many professionals with different specialisations. Therefore, hospitals must implement measures and strategies to ensure structured communication processes among clinicians, clear guidelines and procedures for communicating deteriorations and creating a safety culture promoting teamwork. The widely accepted and internationally adopted method for early detection of deteriorating patients is the implementation of the Early Warning Scores (EWS) systems [10]. EWS systems provide a standardised and systematic approach to patient assessment therefore enhancing patient safety by enabling timely interventions and reducing the risk of adverse events due to unnoticed deterioration Furthermore, the systems often come with alert mechanisms that notify healthcare teams about deteriorating patient thus improves communication and collaboration among clinicians, ensuring that appropriate actions are taken promptly [5, 6, 12, 77, 78]. Given the advantages of the EWS systems, this study therefore illuminates the importance of implementation of the EWS systems in the hospitals.

### Study limitations

This study utilised data from medical records reviews and interviews. Our findings, particularly those drawn from in-depth interviews, may have been affected by social desirability bias. However, we attempted to offset this by including a review of documents to triangulate self-reported findings related to documentation practices by the nurses. Further, conducting the study in three hospitals was another form of triangulation. We did not find significant differences across the study hospitals regarding documentation practices and identifying and responding to deteriorating patients. Carrying out the study in two counties limits the generalisability of our findings. However, we have provided an adequate description of the study settings, particularly the study hospitals. We believe these findings can support readers to exercise judgement in determining the transferability of our study findings. Another limitation of the study was that it did not consider impact of high workload as a contributing factor to the quality of recorded vital signs in patient files. This may play a role in the quality of recorded vital signs but was not thoroughly examined in this research. Therefore, the findings should be interpreted within the context of this limitation, and future studies should consider a more comprehensive approach to understanding the multifaceted nature of this problem.

## Conclusion

Nurses have a critical role in recognising and responding to deteriorating patients. However, this study has demonstrated that nurses did not consistently monitor and document vital signs. Further, these nurses worked in sub-optimal ward environments characterised by inadequate and poorly functioning monitoring equipment; high workload because of staff shortages; sub-optimal communication during emergencies; gaps in teamwork and little training on actions to take when patients deteriorate at the ward level. All these features can negatively affect patient safety, quality of care, and patient outcomes at the ward level. Paying closer attention to the context in which nurses provide care can help to support nurses in promptly identifying and responding to deteriorating patients. Some hospital-level actions that could be taken towards this include providing adequate equipment for monitoring patients; simplifying the chain of communication when patients deteriorate and ensuring awareness of how such communication should be done; providing training targeted at nurses and other cadres of healthcare workers at ward level on collaborative practices including specific packages such as BLS and ACLS.

This study also highlights a broad health system challenge beyond the influence of nurse managers at the ward and hospital level, that of the health worker shortage. The severe shortages reported in this study could hinder safe patient care. They also illustrate a need for policies and strategies that combine short, medium, and long-term approaches to attracting and retaining ward-level healthcare workers. Important considerations include the skill mix of nurses, provisions for conducting a rational review of staffing norms with the input of nursing managers, and engagement with training institutions to have more nurses in the pipeline.

### Supplementary Information

**Supplementary Material 1. **

## Data Availability

The data that support the findings of this study are available from the corresponding author on reasonable request.

## Abbreviations

ACLS: Advanced Cardiac Life Support
BLS: Basic Life Support
BP: Blood pressure
BVM: Bag Valve Mask
CCU: Critical Care Unit
CPR: Cardiopulmonary Resuscitation
HCWs: Healthcare Workers
HDU: High Dependency Unit
HR: Heart rate
ICU: Intensive Care Unit
LOC: Level of consciousness
MO: Medical Officer
PPE: Personal Protective Equipment
RR: Respiratory rate
Sp02: Oxygen saturation
Temp: Temperature
