# Supplementary material for: General ward nurses detection and response to clinical deterioration in three hospitals at the Kenyan coast: a convergent parallel mixed methods study
Source: BMC Nurs. 2024 Mar 1;23:143. doi: 10.1186/s12912-024-01822-2 (PMC10905788; doi:10.1186/s12912-024-01822-2)
Supplement: Supplementary file 1 — Supplementary Material 1. [file 12912_2024_1822_MOESM1_ESM.docx]

**KEY INFORMANT INTERVIEW GUIDE – NURSE WARD AND HOSPITAL MANAGERS**

**Introduction**

Thank you for agreeing to speak with us today about your ward. We would like to learn about the recognition of patient deterioration and actions taken to respond in your ward including what factors or hinder prompt action when a patient’s status starts to decline. This conversation will take about 30-40 minutes of your time. With your permission we would like to record this discussion to enable us to write up our findings.

**Background**

Gender of the respondent:

No of years as a nurse:

No. of years in nurse manager role:

No. of years as in-charge of the current ward:

No. of nurses in the ward:

1. In your ward, what kind of support and resources are available to assist the nurses to quickly identify and respond to a patient whose condition is deteriorating? *Probe for availability and policies and guidelines seeking examples, training opportunities for nurses, availability of equipment and whether sufficient?* Regarding guidelines (if mentioned, how frequently are these reviewed? What are your views on support from your supervisors e.g. head of department, hospital matron, medical superintendent regarding patient safety? *(If supportive, how have they demonstrated this support? If not, what makes the respondent say so?*

2. What proportion of nurses have received BLS/ACLS training after their posting into your ward? How frequent are training refreshers done? How do you decide which nurses to send for training?

3. What is the existing communication protocol for a patient who the nurse in your ward identifies to be deteriorating? *(probe for mode of communication, who is called, and what information is required to be communicated, application of Situation, Background, Assessment, Recommendation (SBAR)* Would you give an example that stands out to you of when a patient’s case was handled well? What about a situation where a patient’s deterioration was not handled well, why do you think this happened? What kinds of actions were taken afterwards to improve?

4. What kinds of challenges exist in your ward that hinder you (and your nurses) from quickly identifying and responding to a patient who is deteriorating? *(probe for an example of a case where identification was delayed)* What action if any have you been able to take in response to these challenges?

5. In your experience, are there certain shifts, or certain seasons when there are more cases of delayed identification and/or response? Why do you think this is the case during these times? What do you think can be done to improve during these times?

**KEY INFORMANT INTERVIEW GUIDE-WARD NURSES**

**Introduction**

Thank you for agreeing to speak with us today about your ward. We would like to learn about the recognition of patient deterioration and actions taken to respond in your ward including what factors or hinder prompt action when a patient’s status starts to decline. This conversation will take about 30-40 minutes of your time. With your permission we would like to record this discussion to enable us to write up our findings.

**Background**

Gender of the respondent:

No of years as a nurse:

No. of years in current ward:

1. In your ward, what kind of support and resources are available to assist the nurses to quickly identify and respond to a patient whose condition is deteriorating? *Probe for availability and policies and guidelines seeking examples, training opportunities for nurses, availability of equipment and whether sufficient?* Regarding guidelines (if mentioned, how frequently are these reviewed? What are your views on support from your colleagues (including doctors) & supervisors e.g. ward-in-charge, regarding patient safety? *(If supportive, how have they demonstrated this support? If not, what makes the respondent say so?*

2.When did you last attend BLS/ACLS training? Have you had refresher training since then? If not, why is that?

3. What is the existing communication protocol when you identify a patient in the ward to be deteriorating? *(probe for mode of communication, who is called, and what information is required to be communicated, application of Situation, Background, Assessment, Recommendation (SBAR)* Would you give an example that stands out to you of when a patient’s case was handled well? What about a situation where a patient’s deterioration was not handled well, why do you think this happened? What kinds of actions were taken afterwards to improve?

4. What kinds of challenges exist in your ward that hinder you (and fellow nurses) from quickly identifying and responding to a patient who is deteriorating? *(probe for an example of a case where identification was delayed)* What action if any have you been able to take in response to these challenges?

5. In your experience, are there certain shifts, or certain seasons when there are more cases of delayed identification and/or response? Why do you think this is the case during these times? What do you think can be done to improve during these times?
